# Does Poorer Pulmonary Function Accelerate Arterial Stiffening? A Cohort Study With Repeated Measurements of Carotid-Femoral Pulse Wave Velocity

**DOI:** 10.1161/HYPERTENSIONAHA.119.13183

**Published:** 2019-08-05

**Authors:** Masaki Okamoto, Martin J. Shipley, Ian B. Wilkinson, Carmel M. McEniery, Carlos A. Valencia-Hernández, Archana Singh-Manoux, Mika Kivimaki, Eric J. Brunner

**Affiliations:** 1 From the Department of Epidemiology and Public Health, University College London, United Kingdom (M.O., M.J.S., C.A.V.-H., A.S.-M., M.K., E.J.B.); 2 Department of Public Health, Graduate School of Medicine, The University of Tokyo, Japan (M.O.); 3 Division of Experimental Medicine and Immunotherapeutics, University of Cambridge, United Kingdom (I.B.W., C.M.M.); 4 Inserm U1153, Epidemiology of Ageing and Neurodegenerative diseases, Paris, France (A.S.-M.).

**Keywords:** epidemiology, inflammation, longitudinal study, respiratory function, respiratory function

## Abstract

Whether poorer pulmonary function accelerates progression of arterial stiffness remains unknown as prior observational studies have not examined longitudinal changes in arterial stiffness in relation to earlier pulmonary function. Data (N=5342, 26% female) were drawn from the Whitehall II cohort study. Participants completed repeated assessments of forced expiratory volume in 1 second (FEV_1_, L) and carotid-femoral pulse wave velocity (cf-PWV, m/s) over 5 years. The effect of FEV_1_ on later cf-PWV and its progression was estimated using linear mixed-effects modeling. Possible explanatory mechanisms, such as mediation by low-grade systemic inflammation, common-cause explanation by preexisting cardiometabolic risk factors, and reverse-causation bias, were assessed. Poorer pulmonary function was associated with later higher cf-PWV and its subsequent progression (cf-PWV 5-year change 0.09, 95% CI 0.03–0.17 per SD lower FEV_1_) after adjustment for age, sex, ethnicity, heart rate, and mean arterial pressure. Decrease in pulmonary function was associated with later higher cf-PWV (0.17, 95% CI 0.04–0.30 in the top compared to bottom quartile of decline in FEV_1_). There was no evidence to support mediation by circulating CRP (C-reactive protein) or IL (interleukin)-6. Furthermore, arterial stiffness was not associated with later FEV_1_ after accounting for cardiometabolic status. In conclusion, poorer pulmonary function predicted future arterial stiffness. These findings support pulmonary function as a clinically important risk factor for arterial stiffness and provide justification for future intervention studies for pulmonary function based on its relationship with arterial stiffness.

To improve health management and facilitate cardiovascular disease (CVD) prevention, it is necessary to identify and control cardiovascular risk factors appropriately at an early stage. Among surrogate markers of cardiovascular risk, vascular function, especially aortic stiffness, which can be assessed noninvasively, is a robust predictor of CVD end points and all-cause mortality.^1,2^ The role of pulmonary function in arterial stiffening has been studied less than other risk factors. As many as 1 in 5 of the general population may have abnormal spirometry patterns^3^ and impairment of pulmonary function is a risk factor for incident angina, myocardial infarction, stroke, and CVD mortality,^4–7^ making it important to identify the mechanisms underlying the association between pulmonary function and CVD. Arterial stiffness is among plausible candidates.

Prior observational studies, including 2 prospective studies,^8,9^ showed inconsistent associations between pulmonary function and arterial stiffness, as measured by pulse wave velocity (PWV).^8–14^ Longitudinal changes in PWV in relation to pulmonary function are unclear, and it remains unknown how spirometrically-defined pulmonary function and its change affect later PWV. The mechanisms connecting pulmonary function and arterial stiffness have not been studied.

To address this gap in research, we assessed the cross-sectional and 5-year prospective associations of forced expiratory volume in 1 second (FEV_1_) with carotid-femoral PWV (cf-PWV) and its progression. In addition, we examined mechanisms other than the direct effect of pulmonary function on arterial stiffness: (1) the possible mediating role of low-grade systemic inflammation,^8^ (2) a possible common-cause explanation considering cardiometabolic factors as the underlying causes for both accelerated arterial stiffness and poorer pulmonary function, and (3) a potential reverse causation (arterial stiffness affecting pulmonary function), among middle-aged and older men and women.

## Methods

### Study Population and Design

Data supporting the findings of this study are available from the corresponding author on reasonable request (data sharing policy: https://www.ucl.ac.uk/epidemiology-health-care/research/epidemiology-and-public-health/research/whitehall-ii/).

The Whitehall II study started in 1985 and recruited 10308 London-based civil servants aged 35 to 55 years (6895 men, 3413 women). Details are published.^15^ Participants responded to follow-up health and lifestyle questionnaire surveys and attended clinical examinations every 4 to 5 years. Pulmonary function was assessed using spirometry during clinical examination in 2002/2004, 2007/2009, and 2012/2013. Aortic stiffness was measured using cf-PWV during the 2007/2009 and 2012/2013 examinations. The analytic sample included 5342 participants who had at least 1 measurement of aortic stiffness, of whom 3484 provided cf-PWV measurements on both occasions. Research Ethics Committee approval and written informed consent from each participant were obtained at each study phase.

### Outcome Measure

The cf-PWV measure indexed arterial stiffness. There is an inverse relationship between PWV and vascular compliance; therefore, the pulse wave travels more quickly through arteries with decreased flexibility than elastic ones. The transit time was defined as the pulse wave’s travel time between the carotid and femoral sites in the supine position, captured by applanation tonometry (SphygmoCor, Atcor Medical, Australia) based on ECG monitoring. Thus, cf-PWV (m/s) was calculated by dividing the path length, measured with a tape measure using a standard protocol, by the transit time. At each assessment, cf-PWV was measured twice unless the difference between measurements was >0.5 m/s when a third measurement was taken. The average of the measurements was used in analyses. Extensive analysis shows that within-person change in adiposity during the study did not have an artifactual effect on measurement of path length or on longitudinal change in cf-PWV.^16^

### Main Exposure

Pulmonary function was evaluated through FEV_1_ (L) using flow-sensing spirometry (MicroPlus Spirometer, Micro Medical Ltd, Kent, United Kingdom) in the standing position. The procedure was conducted by trained nurses following a standard protocol.^17^ FEV_1_ was measured ≤5×, and the maximum measurement identified. Body size, especially standing height, is directly proportional to total lung capacity. To correct FEV_1_ for height dependence, we divided by the square of the participant’s standing height and multiplied by the square of the sample average height: women: 1.61 m, men: 1.75 m.^18^ These adjusted values were used in the analyses.

### Covariates

Two systemic inflammatory indicators were measured at the 2002 to 2004 study phase. CRP (C-reactive protein) is an acute phase reactant: a protein produced by the liver in response to induction by IL (interleukin)-6. Serum CRP levels were measured through a high-sensitivity immuno-nephelometric assay in a BN ProSpec nephelometer, and serum IL-6 level was measured through a high-sensitivity ELISA (Dade Behring, Milton Keynes, United Kingdom; R&D Systems, Oxford, United Kingdom, respectively).^19^

Unmodifiable risk factors were the following: sex, age at each phase, and ethnicity (white or not). Modifiable risk factors were body mass index, waist circumference, systolic blood pressure, mean arterial pressure, heart rate, total cholesterol, high-density lipoprotein cholesterol, hemoglobin A1c, smoking status (current smoker or not), antihypertensive medication (current user or not), and diabetic status (diabetic or not). Each item was examined during the 2007/2009 phase. Systolic blood pressure, mean arterial pressure, and heart rate were also assessed in 2012/2013 because these factors, especially mean arterial pressure and heart rate, can affect the cf-PWV measurement by shifting the form of the pulse wave. Follow-up time in years between the clinical screening in 2007/2009 and 2012/2013 was computed and divided by 5 so that a unit change represented a time difference of 5 years.

### Statistical Analysis

Cuzick nonparametric test was used to test for trend of each variable across quartiles of change in FEV_1_ between 2002/2004 and 2007/2009 waves of the study. Linear mixed-effects modeling was used to assess the cross-sectional and longitudinal effects of pulmonary function (2002/2004, 2007/2009, or its change) on arterial stiffness (2007/2009, 2012/2013, or its progression), allowing for subject-specific random effects for both slope and intercept. To examine the association between FEV_1_ and arterial stiffness, the fixed part of the model (ignoring covariate terms) for cf-PWV measured on individual i on occasion k (=2007/2009 or 2012/2013) at time t:


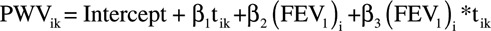


For measurements at 2007/2009, t_ik_=0, and for measurements at 2012/2013, t_ik_=time between 2007/2009 and 2012/2013 measurements. In this model, β_2_ shows the cross-sectional association of FEV_1_ with cf-PWV at 2007/2009, β_1_ shows how cf-PWV changes with time and β_3_, the coefficient for the interaction term, shows how change with time is modified by levels of FEV_1_. Thus, β_3_ indicates whether FEV_1_ is associated with progression of cf-PWV over time after adjusting for the effects of included covariates. In the base model, we adjusted for age, sex, ethnicity, heart rate, and mean arterial pressure. Heart rate and mean arterial pressure were also included as time-varying covariates.

The effect of changes in FEV_1_ before baseline cf-PWV at 2007/2009 and change in cf-PWV between 2007/2009 and 2012/2013 were examined by calculating residuals, regressing FEV_1_ at 2007/2009 on FEV_1_ at 2002/2004. These residuals were included in the mixed models for cf-PWV as a continuous variable and in quartiles. The advantage of using residuals, rather than absolute changes in FEV_1_, is that these residuals are orthogonal to, and independent of, baseline FEV_1_ (2002/2004). Modeling absolute changes with baseline adjustment is a questionable method.^20^ The mixed-effect models were estimated using maximum likelihood. Likelihood-ratio tests were conducted to test for effect modification using fixed-effect interaction terms between pulmonary function quartiles and follow-up time.

To explain the relationship between FEV_1_ and cf-PWV in terms of a hypothesized process, we estimated and tested the indirect effect of FEV_1_ on cf-PWV and its mediation by inflammation (CRP and IL-6) using the Baron and Kenny approach.^21^ CIs for the percentage attenuation were computed using the bootstrap method with 2000 resamplings.

To investigate reverse causation, linear regression models were fitted to estimate the effect of cf-PWV at 2007/2009 on FEV_1_ at 2012/2013. Percentage change after adjustment estimated the role of cardiometabolic factors as a common cause of arterial stiffness and poorer pulmonary function. A sensitivity analysis was conducted to examine potential bias arising from chronic respiratory diseases. We excluded 623 participants (11.7%) at the 2007/2009 phase with diagnosis of chronic obstructive pulmonary disease, asthma, chronic bronchitis, pulmonary fibrosis, pneumoconiosis, interstitial pneumonia, pulmonary tuberculosis, or lung cancer.

We imputed missing data using multiple imputation methods whose models included all assessments related to participants’ vascular function together with risk factors at the 2007/2009 phase. The following auxiliary variables, associated with ≥1 variables with missing data, were included in the imputation models: systolic blood pressure, mean arterial pressure, heart rate, and forced vital capacity from adjacent phases, triglycerides, angina, myocardial infarction, stroke, CVD medication, chronic respiratory diseases, and alcohol dependence. We generated 20 imputed data sets and combined the results using Rubin rules.^22^ Statistical significance was defined as a 2-tailed *P*<0.05. Analyses were conducted using Stata/MP Ver. 13.1 (StataCorp, TX).

## Results

### Sample Characteristics

Participants (N=5342) were mean age 65.4 years in 2007/2009. The cohort was 26% female and of predominantly white ethnic origin. Table 1 shows participant characteristics at baseline stratified according to quartiles of change in pulmonary function between 2002/2004 and 2007/2009. Participants who showed greater decline of FEV_1_ were, on average, older, male, white ethnicity, and had higher cf-PWV at the start of the follow-up. Greater decline in FEV_1_ was associated with being a current smoker, higher waist circumference, higher systolic blood pressure, higher mean arterial pressure, and lower heart rate. There were no associations with body mass index, total cholesterol, or hemoglobin A1c. Medication history of hypertension and diabetes mellitus were not associated with change in FEV_1_.

**Table 1.**
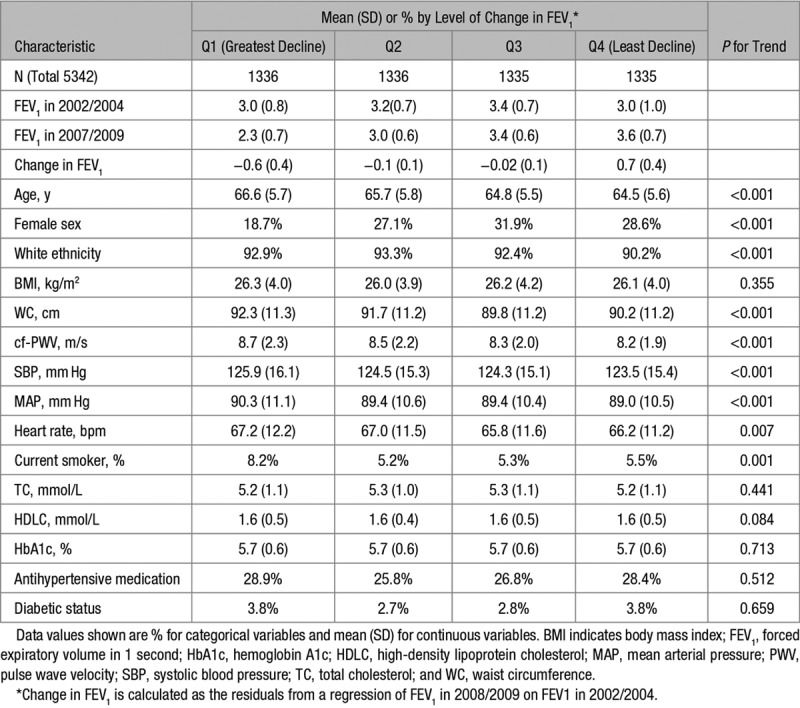
Characteristics of the Study Participants in 2007/2009 by Quartiles of FEV_1_ Changes Between 2002/2004 and 2007/2009

### Effect of Pulmonary Function on Later Arterial Stiffness

Table 2 shows the results of the mixed-effects regression to estimate the effect of pulmonary function (FEV_1_ in 2007/2009, FEV_1_ in 2002/2004, and ΔFEV_1_ between 2002/2004 and 2007/2009) on aortic stiffness (cf-PWV in 2007/2009) and its subsequent progression (Δcf-PWV between 2007/2009 and 2012/2013). Lower FEV_1_ in 2007/2009 was associated cross-sectionally with higher cf-PWV in 2007/2009 after adjustment for age, sex, ethnicity, mean arterial pressure, and heart rate (first panel of Table 2). Cf-PWV was 0.71 m/s (95% CI, 0.52–0.91) higher for those in the highest versus lowest quartile of FEV_1_, and a 1 SD lower FEV_1_ was associated with a 0.28 m/s higher cf-PWV. The right-hand side of the first panel of Table 2 shows that participants in the highest, compared to the lowest, quartile of FEV_1_ in 2007/2009 had slower subsequent progression of cf-PWV between 2007/2009 and 2012/2013 after adjusting for other variables. A 1 SD lower FEV_1_ in 2007/2009 was associated with a subsequent faster cf-PWV progression.

**Table 2.**
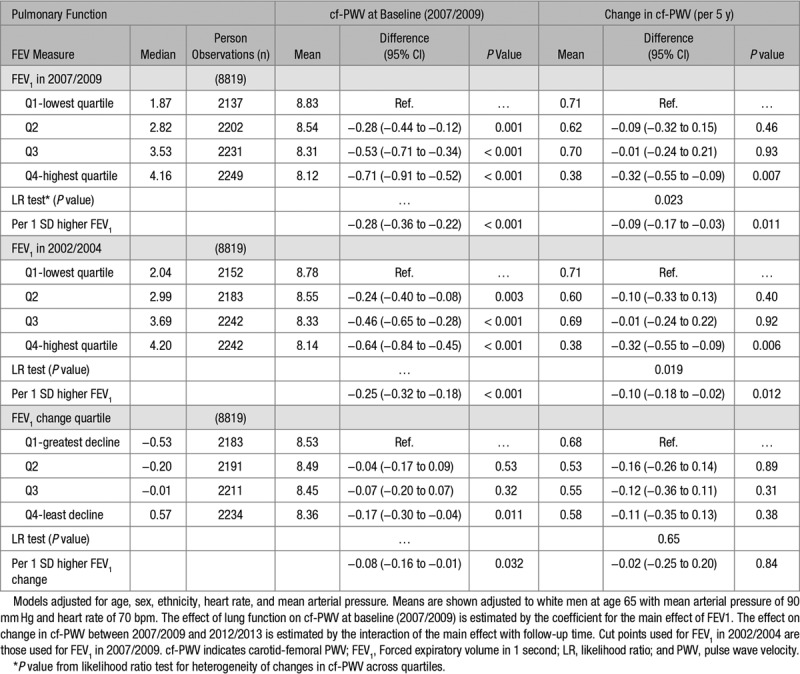
Association Between FEV_1_ (L) cf-PWV (m/s) During the Follow-Up Period

Similarly, as shown in the second panel of Table 2, lower FEV_1_ measured 5 years earlier in 2002/2004 was associated with higher cf-PWV in 2007/2009 and faster progression between 2007/2009 and 2012/2013. Furthermore, in the third panel, decrease in FEV_1_ between 2002/2004 and 2007/2009 was associated with higher subsequent cf-PWV in 2007/2009. Changes in FEV_1_ between 2002/2004 and 2007/2009 were not associated with progression of cf-PWV between 2007/2009 and 2012/2013 (heterogeneity *P*=0.65 across quartiles, trend *P*=0.84).

As sensitivity analyses, the mixed-effect regressions were repeated after excluding participants with prevalent chronic respiratory diseases. The same associations were observed, with lower FEV_1_ in 2007/2009 or 2002/2004 associated with higher cf-PWV in 2007/2009 and faster progression of cf-PWV. As previously described, whereas longitudinal decreases in FEV_1_ between 2002/2004 and 2007/2009 were significantly associated with higher subsequent cf-PWV in 2007/2009, FEV_1_ change (2002/2004 to 2007/2009) was not associated with subsequent progression of cf-PWV.

### Inflammatory Pathway Mediation Analysis

FEV_1_ in 2002/2004 was associated cross-sectionally with CRP and IL-6 (*P*<0.001). The mediation effect of inflammatory markers on the association between FEV_1_ in 2002/2004 and cf-PWV in 2007/2009 is shown in Table 3. CRP in 2002/2004 was not associated with cf-PWV in 2007/2009, and the association of FEV_1_ in 2002/2004 with cf-PWV 2007/2009 was not attenuated after CRP adjustment. IL-6 in 2002/2004 was weakly associated with cf-PWV in 2007/2009 (*P*=0.013). IL-6 adjustment did not attenuate the association between FEV_1_ and cf-PWV. Similarly, adjustment for smoking status had negligible effect on this association (attenuation 0.01%; 95% CI, −5.1 to 5.2).

**Table 3.**
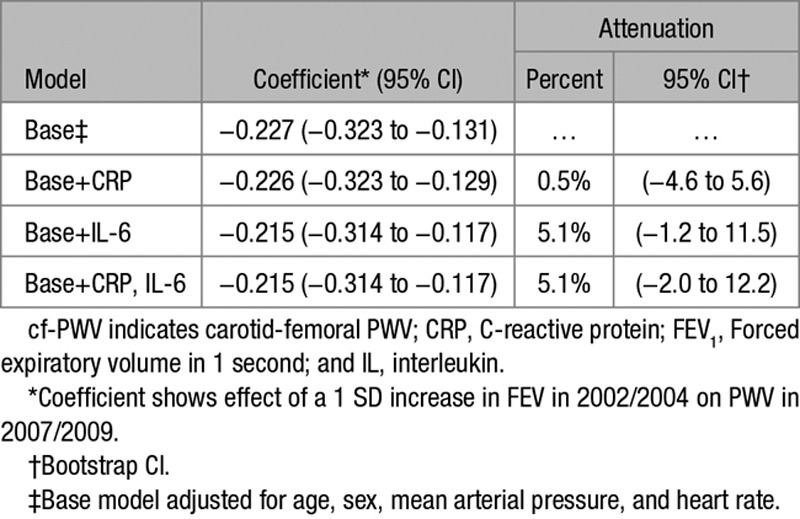
Mediation of the Association Between FEV_1_ in 2002/2004 and cf-PWV in 2007/2009 by Circulating Inflammatory Factors

### Reverse Causation

The association between cf-PWV in 2007/2009 and FEV_1_ in 2012/13 was examined before and after adjustment for cardiometabolic status (Table 4). In the base model, higher cf-PWV in 2007/2009 was weakly associated with poorer FEV_1_ in 2012/2013. Cardiometabolic factors (systolic blood pressure, body mass index, waist circumference, total cholesterol, hemoglobin A1c, and smoking status) were added to the model. The association between cf-PWV and later FEV_1_ was not attenuated significantly, although the PWV-FEV_1_ association was not significant (*P*=0.053) in the adjusted model.

**Table 4.**
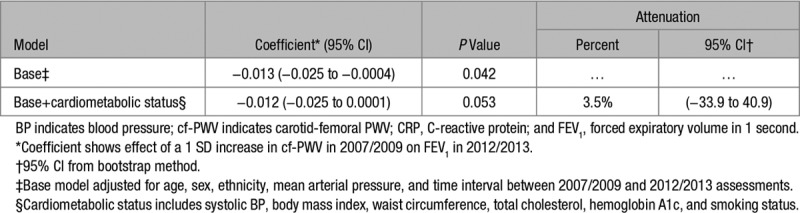
Reverse Causation—Associations of cf-PWV in 2007/2009 With FEV_1_ in 2012/2013

## Discussion

This large longitudinal study provides new evidence for poor pulmonary function as a risk factor for aortic stiffness in early old age. The present findings show that (1) lower FEV_1_ was associated with later higher cf-PWV and its subsequent longitudinal progression after adjustment for age, sex, ethnicity, heart rate, and mean arterial pressure; (2) 5-year decrease in FEV_1_ was associated with higher cf-PWV, whereas the FEV_1_ decrease was not associated with progression of aortic stiffness; (3) there was little evidence that the association between poorer pulmonary function and later higher aortic stiffness was mediated by circulating inflammatory markers CRP and IL-6; and (4) higher cf-PWV was only weakly associated with later impairment of FEV_1_ after adjustment for cardiometabolic status, suggesting that there was no strong evidence for reverse causation. The current evidence supports the clinical importance of observing pulmonary function as a new marker or potential cause for aortic stiffness.

We hypothesized that the association between poorer pulmonary function and higher arterial stiffness would be mediated by low-grade systemic inflammation. The human respiratory system is thought to be particularly vulnerable to environmental factors because it has a large interface with the outside world. Exposure to environmental factors can cause poorer pulmonary function with acute or chronic respiratory inflammation. This could result in low-grade systemic inflammation, which can be measured through higher blood concentration of CRP and IL-6.^23^ Systemic inflammation might contribute to, or trigger, the process of remodeling within vascular walls of arteries, resulting in elastic fibers’ degradation and their replacement by collagen deposition.^24–26^ Studies focusing on low-grade systemic inflammation have typically assessed only CRP. Despite CRP being a general marker of inflammatory processes, IL-6 may be a more sensitive and appropriate marker of systemic inflammation through regulation of CRP synthesis and assumed causal association with cardiovascular end points, such as myocardial infarction.^27^ However, research on the relationship between IL-6 and lung function is also scarce.^28–30^ Our mediation analysis showed that the association between poorer pulmonary function and higher arterial stiffness was unchanged when CRP was used as marker of inflammation and insignificantly mediated by only 5.1% (95% CI, −1.2 to 11.5) when IL-6 was used. Therefore, we found little evidence that the effect of FEV_1_ on cf-PWV could be attributed to mediation by low-grade systemic inflammation, despite the plausibility of this pathophysiological mechanism. This finding was supported by sensitivity analysis, excluding people with chronic respiratory disease.

Analyses to investigate reverse causation were included in this study. Higher cf-PWV was a predictor of later lower FEV_1_, but the association was weak. After controlling for cardiometabolic factors, the cf-PWV- FEV_1_ association was not significant. Two inferences can be made. First, cardiometabolic status does not seem to play an important role as a common cause of higher arterial stiffness and poorer pulmonary function. Second, the prospective association may be due entirely to confounding by cardiometabolic status. In summary, the evidence that aortic stiffness is a determinant of pulmonary function in our cohort is weak.

The balance of our findings suggests pulmonary function may have direct impact on arterial stiffness. Poor pulmonary function may lead to an imbalance between demand and supply of oxygen in the arterial wall to cause aortic stiffening. Development of atherosclerotic lesions may contribute concurrently to arterial stiffening.^31,32^ Alternatively, the association between pulmonary function and aortic stiffness could partly be due to shared structural and mechanical properties of the 2 organs. Elastin may be important in this context, as a protein conferring distensibility and elastic recoil to connective tissue in the aortic wall and lung. Serine proteases and matrix metalloproteinases are able to degrade elastin, contributing to loss of elasticity and potentially to correlated properties of aorta and lung.^8,11,33^

We did not find evidence to support an effect of longitudinal changes in pulmonary function on later cf-PWV change, possibly because the study lacked statistical power. If direct impact of pulmonary function on arterial stiffness is assumed, then, in theory, there should be a significant association between change in FEV_1_ and later change in cf-PWV. However, the mean change in cf-PWV over about 5 years (between 2007/2009 and 2012/2013) was <0.7 m/s; hence, the study may be underpowered to detect such a small effect.

The main strength of this study lies in its longitudinal design, including repeated measurements of pulmonary function and arterial stiffness over time, which enabled us to address causality. To our knowledge, this is the first study to assess the impact of longitudinal changes in FEV_1_ on later aortic stiffness and its progression in early old age. Three statistical approaches were applied to examine the direct effect, possible pathway, and reverse causation. Another strength is the large study sample which included both men and women. This is one of the largest observational studies examining the association between pulmonary function and arterial stiffness, which enables us not only to have good statistical power but also to validate the reference value of FEV_1_ and to avoid spurious differences because of sampling error.

Regarding this study’s limitations, unmeasured confounding factors might also affect the association between pulmonary function and arterial stiffness. For example, genetic factors or susceptibility might contribute to spurious associations.^6,34,35^ Simultaneous loss of flexibility in the connective tissue of both alveoli and arterial walls due to genetic factors could plausibly lead to both impaired pulmonary function and higher arterial stiffness because elastin is known to confer elasticity to both organs. In the context of common origins, the direct effect of FEV_1_ on cf-PWV captured in the current study cannot be separated from the effects of genetic factors or shared pathological mechanisms. Furthermore, the Developmental Origins of Health And Disease hypothesis demonstrates that adverse exposures in early life could lead to permanent structural and metabolic changes, resulting in both pulmonary and CVDs in later life.^36,37^ If impaired pulmonary function and higher arterial stiffness share early life origins, it will be also necessary to keep in mind that early life prevention could be indicated, although the evidence currently available for the shared origins is limited. In addition, within-person longitudinal variability of inflammatory markers may bias attenuation towards the null in the mediation analysis,^38^ although recent analyses, based on the same cohort study, examining IL-6 and CRP have suggested such variability could be a relatively minor concern.^39,40^

Finally, the data used in this study were from middle- to older-aged, white-collar, London-based civil servants; therefore, the generalisability of this study’s findings need to be confirmed in future research including other generations, ethnicities, and occupation types. In the context of standard risk factor-CVD associations, despite marked differences in both risk factors and disease incidence, close agreement between the findings from Whitehall II and general population samples have been found.^41^

## Perspective

The present findings suggest that pulmonary function could have predictive value for future increases in arterial stiffness and its progression. To clarify the causal effect of pulmonary function measured by FEV_1_ on later aortic stiffness, a possible indirect pathway through mediation by inflammation and reverse causation considering cardiometabolic status were investigated. These results imply that the association between poorer pulmonary function and later increase in aortic stiffness is not mediated by systemic inflammation indicated by elevated levels of CRP and IL-6. Furthermore, higher cf-PWV does not predict later impairment of FEV_1_ after accounting for cardiometabolic status.

Future intervention studies are needed to clarify the preventive effect of monitoring and intervening in lung function on aortic stiffness progression. The current evidence supports the clinical importance of observing pulmonary function as a new potential cause for aortic stiffness. Considering the feasible nature of spirometry as a routine clinical examination, FEV_1_ may be an effective potential option for improving health management and facilitating CVD prevention.

## Acknowledgments

We thank every participant in the Whitehall II cohort study, as well as all Whitehall II researchers and support staff who make the study possible.

## Sources of Funding

The Whitehall II study is supported by the British Heart Foundation (RG/16/11/32334), UK Medical Research Council (K013351) and US National Institute on Aging (R01AG013196; R01AG034454; R01AG056477). M. Kivimaki is additionally supported by the UK Medical Research Council (S011676), NordForsk (the Nordic Research Programme on Health and Welfare), the Academy of Finland (311492), and Helsinki Institute of Life Science.

## Disclosures

None.

## Supplementary Material

## Novelty and Significance

### What Is New?

- This article provides evidence on the predictive importance of poor lung function in relation to future increases in arterial stiffness and its progression among men and women, accounting for possible explanatory mechanisms.

### What Is Relevant?

- The current findings support the clinical value of observing lung function as a new marker or potential cause for vascular aging, which is relevant to hypertension and its related diseases.

### Summary

Poor lung function predicted future arterial stiffness. This supports lung function as a clinically important risk factor for arterial stiffness and provides justification for future intervention studies for lung function based on its relationship with arterial stiffness.
