# Supplementary figures and images for: Does Poorer Pulmonary Function Accelerate Arterial Stiffening? A Cohort Study With Repeated Measurements of Carotid-Femoral Pulse Wave Velocity
Source: Hypertension. 2019 Aug 5;74(4):929–35. doi: 10.1161/HYPERTENSIONAHA.119.13183 (PMC6756258; doi:10.1161/HYPERTENSIONAHA.119.13183)

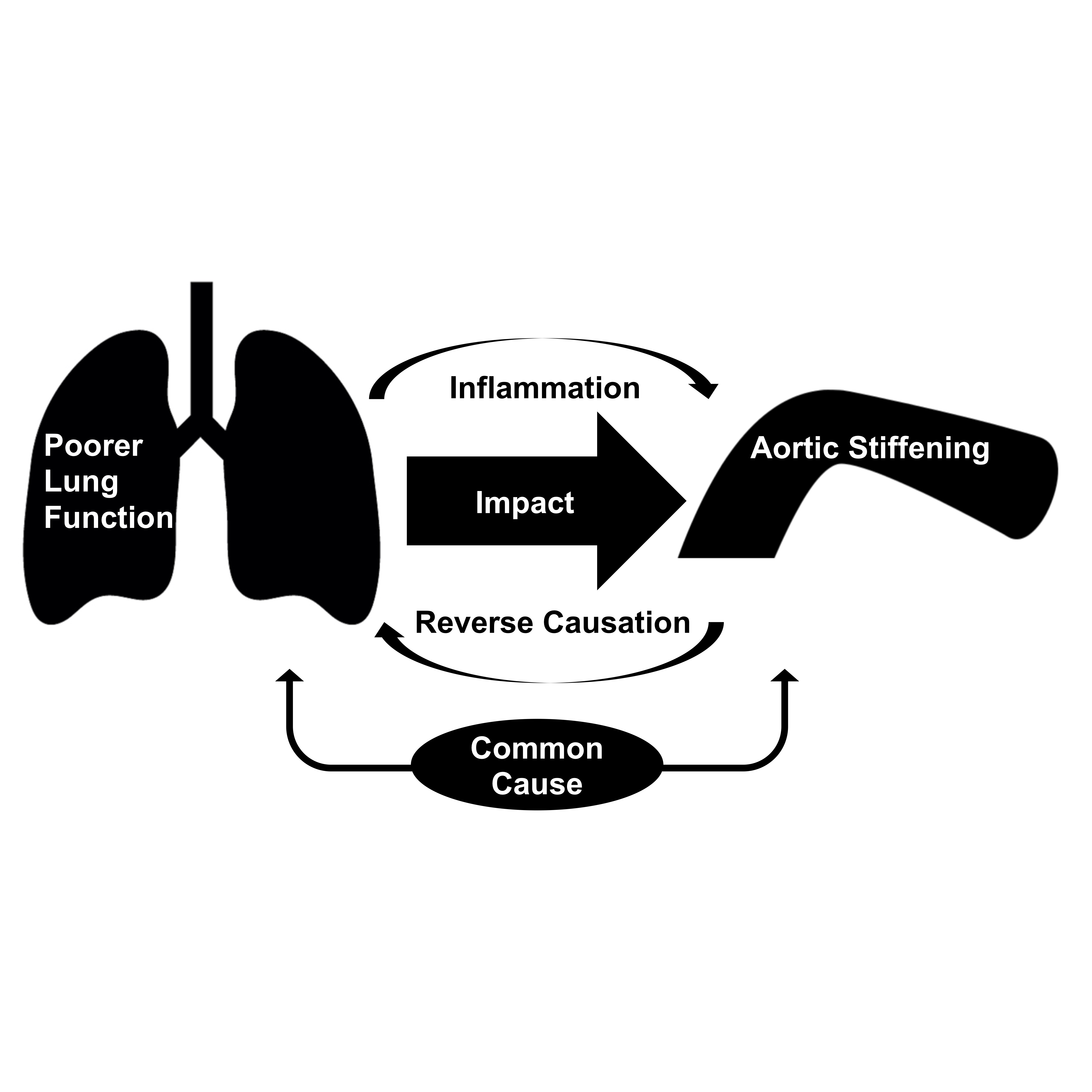

Supplement: Supplementary file 1 [file hyp-74-0929-s001.jpg]
